# 急性髓系白血病不同性别间的治疗结局分析

**DOI:** 10.3760/cma.j.cn121090-20260312-00129

**Published:** 2026-08

**Authors:** 宗儒 李, 露 于, 利娟 胡, 岩 尉, 文冰 段, 婷 赵, 强 付, 浩 江, 菲菲 唐, 婧 王, 晋松 贾, 于谦 孙, 晓冬 莫, 兰平 许, 昱 王, 晓辉 张, 晓军 黄, 倩 江

**Affiliations:** 1 北京大学人民医院，北京大学血液病研究所，国家血液系统疾病临床医学研究中心，造血干细胞移植北京市重点实验室，北京 100044 National Clinical Research Center for Hematologic Disease, Peking University Institute of Hematology, Peking University People's Hospital, Beijing 100044, China; 2 北京大学人民医院青岛医院，青岛 266100 Peking University People's Hospital Qingdao Hospital, Qingdao 266100, China

**Keywords:** 白血病，髓系，急性, 性别, 治疗结局, Leukemia, myeloid, acute, Sex, Treatment outcome

## Abstract

**目的:**

比较急性髓系白血病（AML）患者中男性和女性的治疗结局。

**方法:**

本研究为回顾性队列研究。收集2017年1月至2025年6月在北京大学人民医院新诊断的成人AML患者的数据。主要观察终点包括完全缓解伴或不伴血液学恢复（CR/CRi）率和总生存（OS）率。

**结果:**

纳入2 264例患者，1 180例（52％）为男性。男性的血红蛋白、尿酸、白蛋白水平比女性更高，估算肾小球滤过率更低（白蛋白*P*＝0.006，余均*P*<0.001）。男性携带髓系基因突变的个数比女性更多（*P*＝0.001），且携带ASXL1、U2AF1、SRSF2、RUNX1、ZRSR2、DDX41、SETBP1、TET2和FLT3-TKD突变，以及−7/7q−和+8的比例较女性更高，携带NPM1、DNMT3A和FLT3-ITD突变的比例更低，差异均有统计学意义（均*P*<0.05）。多因素分析显示，男性与更低的1个疗程CR/CRi率（*OR*＝0.80，95％*CI*：0.66～0.97，*P*＝0.023），以及更低的无复发生存（RFS）率（*HR*＝1.17，95％*CI*：1.00～1.37，*P*＝0.046）和OS率（*HR*＝1.27，95％*CI*：1.09～1.47，*P*＝0.002）显著相关。欧洲白血病网（ELN）2022预后良好、中等和不良的患者中，均可见男性较女性的OS率更低。接受阿扎胞苷联合维奈克拉方案的男性患者1个疗程CR/CRi率更低（62％对73％，*P*＝0.004），强化疗组（70％对71％，*P*＝0.593）和CAG组（54％对62％，*P*＝0.222）男女患者的1个疗程CR/CRi率差异均无统计学意义。在获得CR/CRi的患者中，男性4年OS率在首次CR期异基因造血干细胞移植组［74％（95％*CI*：69％～78％）对81％（95％*CI*：77％～85％），*P*＝0.007］和第2次CR或未达CR/CRi移植组［54％（95％ *CI*:43％～66％）对69％（95％ *CI*:58％～80％），*P*＝0.035］均低于女性；未移植组男性的4年OS率有低于女性的趋势［55％（95％ *CI*:49％～60％）对59％（95％ *CI*:54％～64％），*P*＝0.084］。

**结论:**

在AML患者中，男性较女性携带预后不良的基因突变和染色体异常比例更高；性别间治疗反应率和OS率存在差异。

## Abstract (en)

**Objective:**

To compare the treatment outcomes between male and female patients with acute myeloid leukemia（AML）.

**Methods:**

In this retrospective cohort study, data from adult patients with newly diagnosed AML at Peking University People's Hospital between January 2017 and June 2025 were collected and analyzed. The primary outcomes included complete remission with or without incomplete hematological recovery（CR/CRi）and overall survival（OS）rate.

**Results:**

A total of 2 264 patients were enrolled, including 1 180 males（52％）. Males had significantly higher hemoglobin, uric acid, and albumin levels, but a lower estimated glomerular filtration rate than females（*P*＝0.006 for albumin, *P*<0.001 for other variables）. Males harbored a greater number of myeloid gene mutations（*P*＝0.001）and showed significantly higher frequencies of ASXL1, U2AF1, SRSF2, RUNX1, ZRSR2, DDX41, SETBP1, TET2, and FLT3-TKD mutations, as well as −7/7q− and +8 chromosomal abnormalities. Contrarily, males exhibited significantly lower frequencies of NPM1, DNMT3A, and FLT3-ITD mutations than females. Multivariate analysis showed that male sex was significantly associated with a lower rate of CR/CRi after one course of induction therapy（*OR*＝0.80, 95％ *CI*: 0.66–0.97, *P*＝0.023）and inferior relapse-free survival（*HR*＝1.17, 95％ *CI*: 1.00–1.37, *P*＝0.046）and OS（*HR*＝1.27, 95％ *CI*: 1.09–1.47, *P*＝0.002）. Among patients classified as having favorable-, intermediate-, or adverse-risk disease according to ELN 2022 risk classification, males had inferior OS compared with females. In patients receiving the azacitidine plus venetoclax regimen, males had a significantly lower CR/CRi rate after one treatment course（62％ *vs* 73％, *P*＝0.004）, but the difference was not significant in those receiving intensive chemotherapy（70％ *vs* 71％, *P*＝0.593）or the CAG regimen（54％ *vs* 62％, *P*＝0.222）. Among patients who achieved CR/CRi, the 4-year OS rate was significantly lower in males than in females in both the allogeneic hematopoietic stem cell transplantation（allo-HSCT）group with CR_1_［74％（95％ *CI*: 69％–78％）*vs* 81％（95％ *CI*: 77％–85％）, *P*＝0.007］and allo-HSCT group with CR_2_ or no response［54％（95％ *CI*: 43％–66％）*vs* 69％（95％ *CI*: 58％–80％）, *P*＝0.035］. In the non-transplant group, males showed a trend toward inferior 4-year OS rate［55％（95％ *CI*: 49％–60％）*vs* 59％（95％ *CI*: 54％–64％）, *P*＝0.084］.

**Conclusion:**

In patients with AML, males are more likely to harbor myelodysplasia-related gene mutations and chromosomal abnormalities, and sex-based disparities in the treatment response and OS rates have also been observed.

既往的研究发现，接受传统化疗的急性髓系白血病（AML）患者中女性相较于男性有更高的总生存（OS）率[1]–[4]。与女性相比，更高比例的男性携带预后不良的分子遗传学异常，如ASXL1、SRSF2、U2AF1和RUNX1等[5]。在国内，关于性别因素对AML患者预后的影响，尚缺乏大宗病例的系统报道。因此，本研究拟探究性别在不同疾病危险度和不同治疗场景下对AML患者治疗结局的影响。

## 病例与方法

1. 病例：本研究为回顾性队列研究。收集2017年1月至2025年6月在北京大学人民医院新诊断的成人AML患者的数据。所有患者的诊断根据2022版WHO白血病分类标准[6]。本研究纳入了至少接受1个疗程化疗的AML患者，排除了慢性髓细胞性白血病急变期和急性早幼粒细胞白血病患者，以及缺乏完整的细胞遗传学、分子生物学分析和二代测序的患者。本研究已获得北京大学人民医院伦理委员会的批准（批件号：2026PHB182-001）。

2. 免疫表型分析、细胞遗传学和分子生物学分析及二代测序：在患者初次诊断AML时进行免疫表型分析、细胞遗传学和分子生物学分析[7]–[9]。初次诊断时的标本采用Illumina测序平台（美国Illumina公司）进行靶向DNA测序，平均测序深度为2 000×。使用的血液肿瘤基因组合在2021年11月之前包括175个基因，在2021年11月之后扩展为290个基因组合。

3. 治疗方案、疗效评估和结局：诱导化疗前评估患者对强化疗的耐受性。适合强化疗的患者接受HAA（高三尖杉酯碱+阿糖胞苷+阿柔比星）、IA（去甲氧柔红霉素+阿糖胞苷）或DA（柔红霉素+阿糖胞苷）方案，不适合强化疗的患者接受CAG（阿糖胞苷+阿克拉霉素+G-CSF）方案或AZA-VEN（阿扎胞苷+维奈克拉）方案治疗。符合异基因造血干细胞移植（allo-HSCT）适应证的患者在第1次完全缓解（CR_1_）期接受移植，否则继续接受中剂量或大剂量阿糖胞苷强化疗方案或者前述弱化疗方案进行巩固化疗[10]。对于难治或复发的患者，接受FLAG（氟达拉滨、阿糖胞苷、G-CSF）、CLAG（克拉屈滨、阿糖胞苷、G-CSF）或AZA-VEN等方案进行挽救治疗，在CR_2_期接受移植。

本研究以完全缓解伴或不伴血液学恢复（CR/CRi）作为疗效评估终点，评估时间点为1个疗程后和整个化疗期间。危险分层、CR/CRi、累积复发率（CIR）、无复发生存（RFS）期和OS期的判定参照2022版欧洲白血病网（ELN）指南[11]。CIR定义为从达到CR/CRi至末次随访期间累积血液学复发事件的发生率，其中非复发死亡被视为竞争风险。RFS期定义为达到CR/CRi至复发或死亡的时间，OS期定义为诊断至死亡的时间，对于未发生事件或失访的患者，删失在末次随访时间。

4. 随访：本研究采用电话随访，随访的截止时间为2026年4月30日。

5. 统计学处理：连续变量以中位数和四分位数描述，使用Mann-Whitney *U*检验进行比较；分类变量以频数和频率描述，使用Pearson卡方检验或Fisher精确检验进行比较。CIR和累积非复发死亡率使用Fine-Gray检验比较。RFS和OS使用Log-rank检验比较，并使用Kaplan-Meier方法绘制生存曲线。Logistic回归、竞争风险回归和Cox回归用于分析与结局相关的影响因素。*P*值是基于双尾假设检验计算的，其中1型错误率设定为5％。

## 结果

一、患者特征

本研究纳入2 264例患者，基线特征详见表1。其中1 180例（52％）患者为男性，1 084例（48％）患者为女性。中位年龄为47（*IQR*：34～58）岁。419例（19％）患者的Charlson共病指数（CCI）[12]>2，即存在AML以外的合并症。根据2022年ELN指南，预后良好组880例（39％），预后中等组563例（25％），预后不良组821例（36％）。

**表1:** 男性和女性急性髓系白血病（AML）患者特征和治疗方案比较

临床特征	男性（1 180例）	女性（1 084例）	*P*值
年龄［岁，*M*（*IQR*）］	47（34～59）	47（34～58）	0.881
治疗相关或继发白血病［例（％）］	168（14）	135（13）	0.213
CCI>2［例（％）］	227（19）	192（18）	0.351
WBC［×10^9^/L，*M*（*IQR*）］	11（3～35）	10（3～31）	0.324
HGB［g/L，*M*（*IQR*）］	87（68～110）	83（71～99）	<0.001
PLT［×10^9^/L，*M*（*IQR*）］	48（25～101）	53（28～104）	0.102
骨髓原始细胞［％，*M*（*IQR*）］	52（32～71）	53（34～71）	0.111
白蛋白［g/L，*M*（*IQR*）］	42（39～45）	41（38～45）	0.006
尿酸［µmol/L，*M*（*IQR*）］	359（292～441）	271（218～339）	<0.001
估算肾小球滤过率［ml·min^−1^·（1.73 m^2^）^−1^，*M*（*IQR*）］	100（84～112）	104（92～117）	<0.001
总胆红素［µmol/L，*M*（*IQR*）］	11（8～15）	10（8～15）	0.044
ELN2022分层［例（％）］			0.004
预后良好	457（39）	423（39）	
预后中等	264（22）	299（28）	
预后不良	459（39）	362（33）	
诱导化疗方案［例（％）］			0.216
HAA	293（25）	267（25）	
IA/DA	422（36）	408（38）	
CAG	128（11）	126（11）	
AZA-VEN	337（29）	283（26）	
CR/CRi后治疗［例（％）］^a^			0.973
CR_1_期allo-HSCT	396（39）	382（40）	
CR_2_或未达CR期allo-HSCT	95（9）	89（9）	
未行allo-HSCT	519（52）	491（51）	

**注** CCI：Charlson共病指数；ELN：欧洲白血病网；HAA：高三尖杉酯碱+阿糖胞苷+阿柔比星；IA：去甲氧柔红霉素+阿糖胞苷；DA：柔红霉素+阿糖胞苷；CAG：阿糖胞苷+阿克拉霉素+G-CSF；AZA-VEN：阿扎胞苷+维奈克拉；CR/CRi：完全缓解伴或不伴血液学恢复；allo-HSCT：异基因造血干细胞移植；CR_1_：第1次完全缓解。^a^共1 972例患者进行了CR/CRi后治疗，男性1 010例，女性962例

男性患者的血红蛋白、白蛋白、尿酸水平较女性更高，而估算肾小球滤过率较女性更低。男性携带ASXL1、U2AF1、SRSF2、RUNX1、ZRSR2、DDX41、SETBP1、TET2、FLT3-TKD、−7/7q−和+8的比例较女性更高，携带NPM1、DNMT3A和FLT3-ITD突变的比例更低，差异具有统计学意义（均*P*<0.05）（图1）。男性携带基因突变（平均数：2.8对2.6，*P*＝0.001）和骨髓增生异常相关基因突变（平均数：0.52对0.34，*P*＝0.001）的个数显著多于女性。男性患者预后中等的比例更低（22％对28％），预后不良的比例更高（39％对33％），而预后良好的比例和女性患者相似（39％对39％），组间比较差异有统计学意义（*P*＝0.004）。

**图1:**
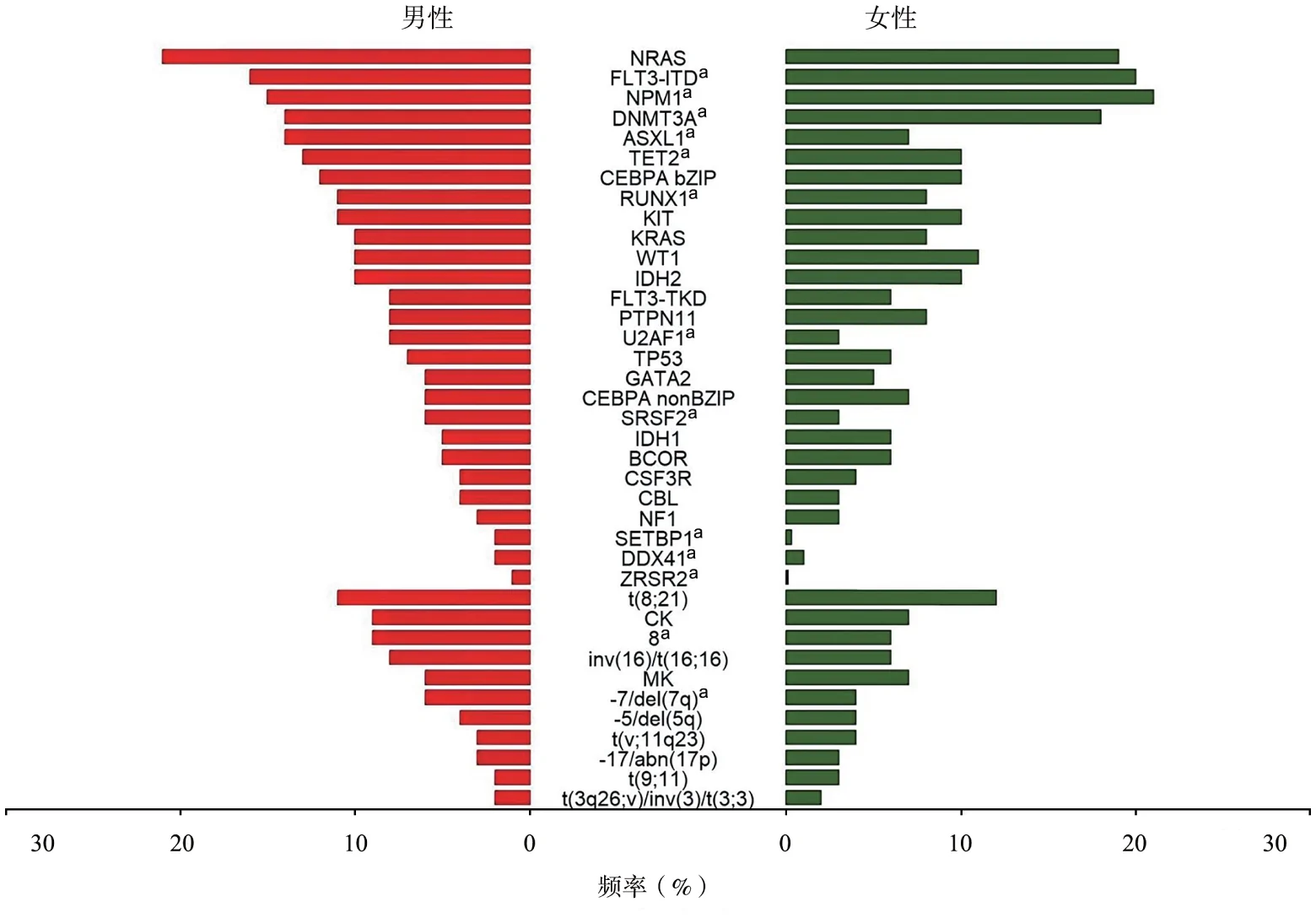
男性和女性急性髓系白血病患者的基因突变和细胞遗传学改变频率比较 **注** CK：复杂核型；MK：单体核型。^a^男性和女性的发生率差异有统计学意义

二、治疗方案

根据诱导化疗方案将患者分为三组：强化疗组1 390例，包括接受HAA方案560例，IA方案706例，DA方案124例；CAG组254例；AZA-VEN组620例。在1 972例达CR/CRi的患者中，CR_1_期接受allo-HSCT 778例，CR_2_或复发后未达CR/CRi接受移植184例。男性和女性患者接受强化疗（61％比63％，*P*＝0.413）和allo-HSCT的比例（CR_1_移植：39％对40％，CR_2_或复发后未达CR移植：9％对9％，*P*＝0.973）差异均无统计学意义。

三、治疗反应和结局

1. 总体人群：本研究中所有患者的中位随访时间为28（*IQR*：13～48）个月，存活者中位随访40（*IQR*：26～58）个月，失访例数为28例（1％）。1个疗程诱导化疗后1 538例（68％）达到CR/CRi，最终1 972例（87％）达CR/CRi。在最终达CR/CRi的患者中683例复发，4年CIR、累积非复发死亡率（NRM）和RFS率分别为35％（95％*CI*：33％～38％）、9％（95％*CI*：7％～10％）和56％（95％*CI*：54％～59％）。男性患者的1个疗程CR/CRi率（66％对70％，*P*＝0.019）和最终CR/CRi率（86％对89％，*P*＝0.025）比女性患者更低，4年CIR［37％（95％*CI*：33％～40％）对34％（95％*CI*：30％～37％），*P*＝0.141］（图2A）和NRM［10％（95％*CI*：8％～12％）对8％（95％*CI*：6％～10％），*P*＝0.177］（图2B）有高于女性的趋势，4年RFS率［54％（95％*CI*：50％～57％）对59％（95％*CI*：55％～62％），*P*＝0.023］（图2C）和OS率［55％（95％*CI*：52％～58％）对63％（95％*CI*：60％～67％），*P*<0.001］（图2D）比女性更低。删失移植后，中位随访时间为10（6～25）个月，男性的2年OS率［62％（95％*CI*：59％～66％）对70％（95％*CI*：66％～74％），*P*<0.001］较女性更低，4年CIR、累积NRM和RFS率差异均无统计学意义。在血液学复发的患者中，男性的4年OS率较女性更低［26％（95％*CI*：21％～31％）对35％（95％*CI*：29％～41％），*P*＝0.006］。

**图2:**
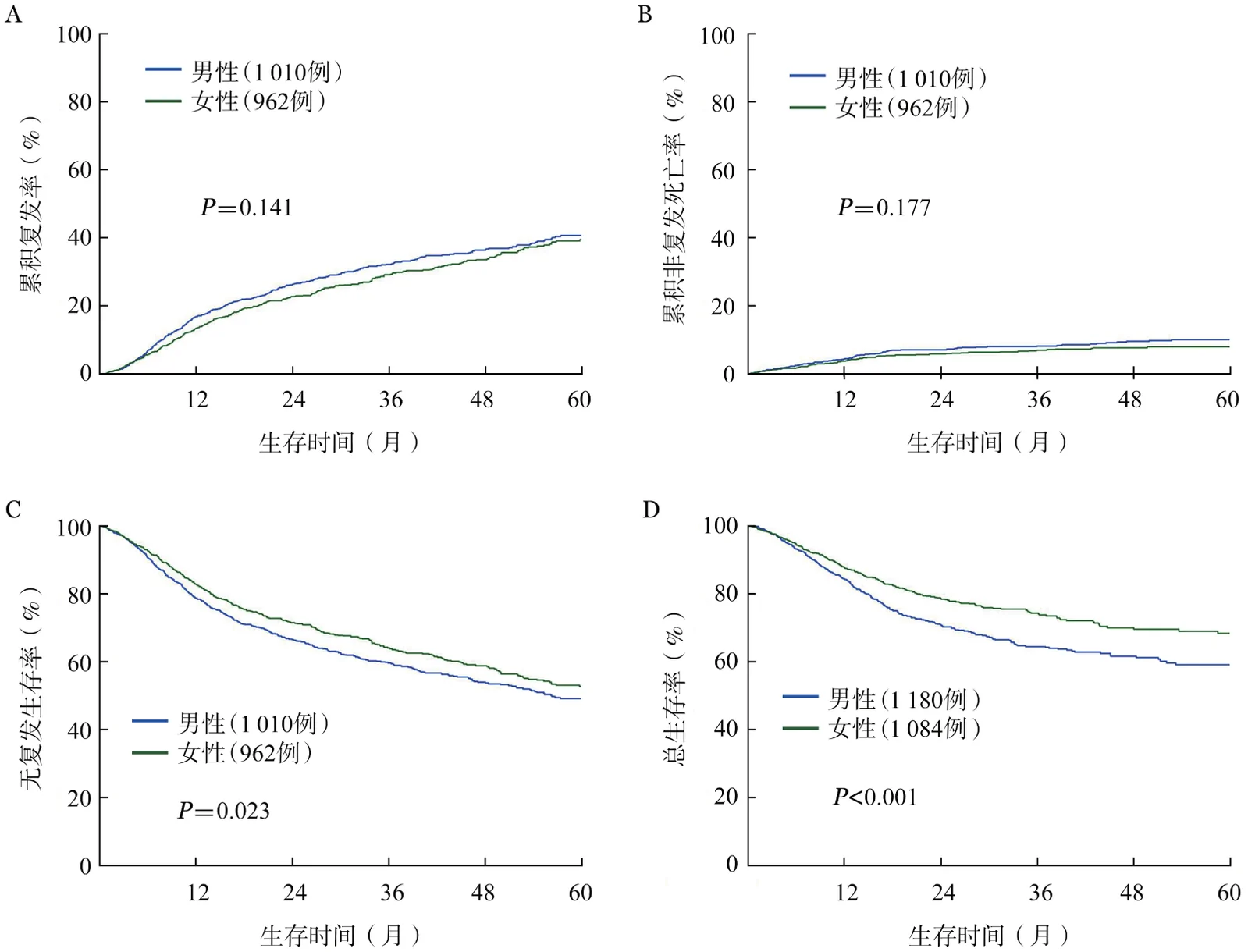
总体人群中男性和女性的累积复发率（A）、累积非复发死亡率（B）、无复发生存率（C）和总生存率（D）比较

多因素分析显示，男性与更低的1个疗程CR/CRi率（*OR*＝0.80，95％ *CI*：0.66～0.97，*P*＝0.023），以及更低的RFS率（*HR*＝1.17，95％ *CI*：1.00～1.37，*P*＝0.046）和OS率（*HR*＝1.27，95％ *CI*：1.09～1.47，*P*＝0.002）显著相关（表2、3）。此外，年龄、治疗相关或继发白血病、白细胞计数、血红蛋白、ELN2022分层和CR_1_期移植等变量与RFS、OS显著相关。

**表2:** 急性髓系白血病患者1个疗程后和最终达CR/CRi的多因素分析

因素	1个疗程后达CR/CRi		最终达CR/CRi	
	*OR*（95％*CI*）	*P*值	*OR*（95％*CI*）	*P*值
男性	0.80（0.66～0.97）	0.023	0.79（0.60～1.03）	0.085
年龄	0.95（0.88～1.02）	0.145	0.94（0.85～1.04）	0.202
治疗相关或继发白血病	0.63（0.48～0.83）	0.001	0.48（0.34～0.67）	<0.001
CCI>2	0.86（0.67～1.10）	0.236	0.76（0.55～1.05）	0.097
WBC	0.98（0.96～0.99）	0.010	0.97（0.95～1.00）	0.040
HGB	1.06（1.02～1.11）	0.003	1.04（0.99～1.10）	0.153
PLT	0.99（0.98～1.00）	0.008	0.99（0.98～1.00）	0.171
骨髓原始细胞			1.00（0.94～1.07）	0.998
ELN2022分层		<0.001		<0.001
预后良好（参照）	1		1	
预后中等	0.37（0.28～0.48）	<0.001	0.20（0.12～0.34）	<0.001
预后不良	0.28（0.22～0.35）	<0.001	0.11（0.07～0.18）	<0.001
化疗方案		0.013		0.009
强化疗（参照）	1		1	
CAG	0.92（0.68～1.25）	0.603	0.71（0.47～1.07）	0.920
AZA-VEN	1.39（1.08～1.78）	0.009	0.61（0.44～0.85）	0.004

**注** 连续变量年龄、WBC、HGB、PLT、骨髓原始细胞的风险比均以每10个单位的变化进行计算。CR/CRi：完全缓解伴或不伴血液学恢复；CCI：Charlson共病指数；ELN2022：2022年欧洲白血病网指南；强化疗：包括高三尖杉酯碱+阿糖胞苷+阿柔比星（HAA）、去甲氧柔红霉素+阿糖胞苷（IA）和柔红霉素+阿糖胞苷（DA）方案；CAG：阿糖胞苷+阿克拉霉素+G-CSF；AZA-VEN：阿扎胞苷+维奈克拉

**表3:** 急性髓系白血病患者累积复发率（CIR）、无复发生存（RFS）率和总生存（OS）率的多因素分析

因素	CIR		RFS		OS	
	*HR*（95％*CI*）	*P*值	*HR*（95％*CI*）	*P*值	*HR*（95％*CI*）	*P*值
男性	1.13（0.94～1.35）	0.190	1.17（1.00～1.37）	0.046	1.27（1.09～1.47）	0.002
年龄	1.10（1.01～1.20）	0.025	1.10（1.03～1.19）	0.009	1.10（1.02～1.18）	0.010
治疗相关或继发白血病	1.30（0.99～1.71）	0.056	1.42（1.12～1.79）	0.004	1.38（1.13～1.69）	0.002
CCI>2	1.07（0.85～1.35）	0.570	1.00（0.82～1.22）	0.986	1.12（0.93～1.34）	0.236
WBC	1.03（1.01～1.05）	0.003	1.02（1.00～1.04）	0.012	1.03（1.01～1.05）	<0.001
HGB	0.95（0.91～0.98）	0.003	0.94（0.91～0.97）	<0.001	0.94（0.91～0.97）	<0.001
骨髓原始细胞	1.02（0.98～1.07）	0.350	1.01（0.97～1.04）	0.804	1.01（0.97～1.04）	0.696
估算肾小球滤过率	1.02（0.97～1.08）	0.410	1.00（0.96～1.05）	0.883	0.96（0.83～1.10）	0.521
白蛋白	1.05（0.89～1.23）	0.600	0.98（0.84～1.13）	0.759	0.99（0.94～1.03）	0.520
ELN2022		<0.001		<0.001		<0.001
预后良好（参照）	1		1		1	
预后中等	1.77（1.40～2.23）	<0.001	1.81（1.48～2.22）	<0.001	2.57（2.07～3.19）	<0.001
预后不良	2.16（1.74～2.69）	<0.001	2.29（1.88～2.79）	<0.001	3.60（2.93～4.41）	<0.001
化疗方案		0.520		0.009		0.080
强化疗（参照）	1		1		1	
CAG	0.88（0.64～1.20）	0.160	1.01（0.78～1.33）	0.920	1.21（0.95～1.54）	0.125
AZA-VEN	1.18（0.94～1.48）	0.410	1.36（1.11～1.67）	0.003	1.23（1.02～1.50）	0.034
CR_1_ allo-HSCT	0.22（0.17～0.28）	<0.001	0.30（0.25～0.37）	<0.001	0.29（0.23～0.35）	<0.001

**注** 连续变量年龄、WBC、HGB、骨髓原始细胞、估算肾小球滤过率的风险比均以每10个单位的变化进行计算。CCI：Charlson共病指数；ELN2022：2022年欧洲白血病网指南；强化疗：包括高三尖杉酯碱+阿糖胞苷+阿柔比星（HAA）、去甲氧柔红霉素+阿糖胞苷（IA）和柔红霉素+阿糖胞苷（DA）方案；CAG：阿糖胞苷+阿克拉霉素+G-CSF；AZA-VEN：阿扎胞苷+维奈克拉；CR_1_ allo-HSCT：第1次完全缓解（伴或不伴血液学恢复）后异基因造血干细胞移植

2. 根据ELN2022危险度分层：在预后良好组中，男性和女性的1个疗程CR/CRi率（82％对85％，*P*＝0.228）、最终CR/CRi率（98％对97％，*P*＝0.854）、4年CIR、累积NRM和RFS率差异均无统计学意义，但男性的4年OS率显著低于女性［73％（95％*CI*：69％～78％）对78％（95％*CI*：74％～83％），*P*＝0.037］（图3A）。复发患者中，男性的4年OS率亦显著低于女性［40％（95％*CI*：31％～49％）对50％（95％*CI*：40％～61％），*P*＝0.029］。多因素分析显示，男性与更低的OS率显著相关（*HR*＝1.41，95％*CI*：1.05～1.90，*P*＝0.023）。

**图3:**
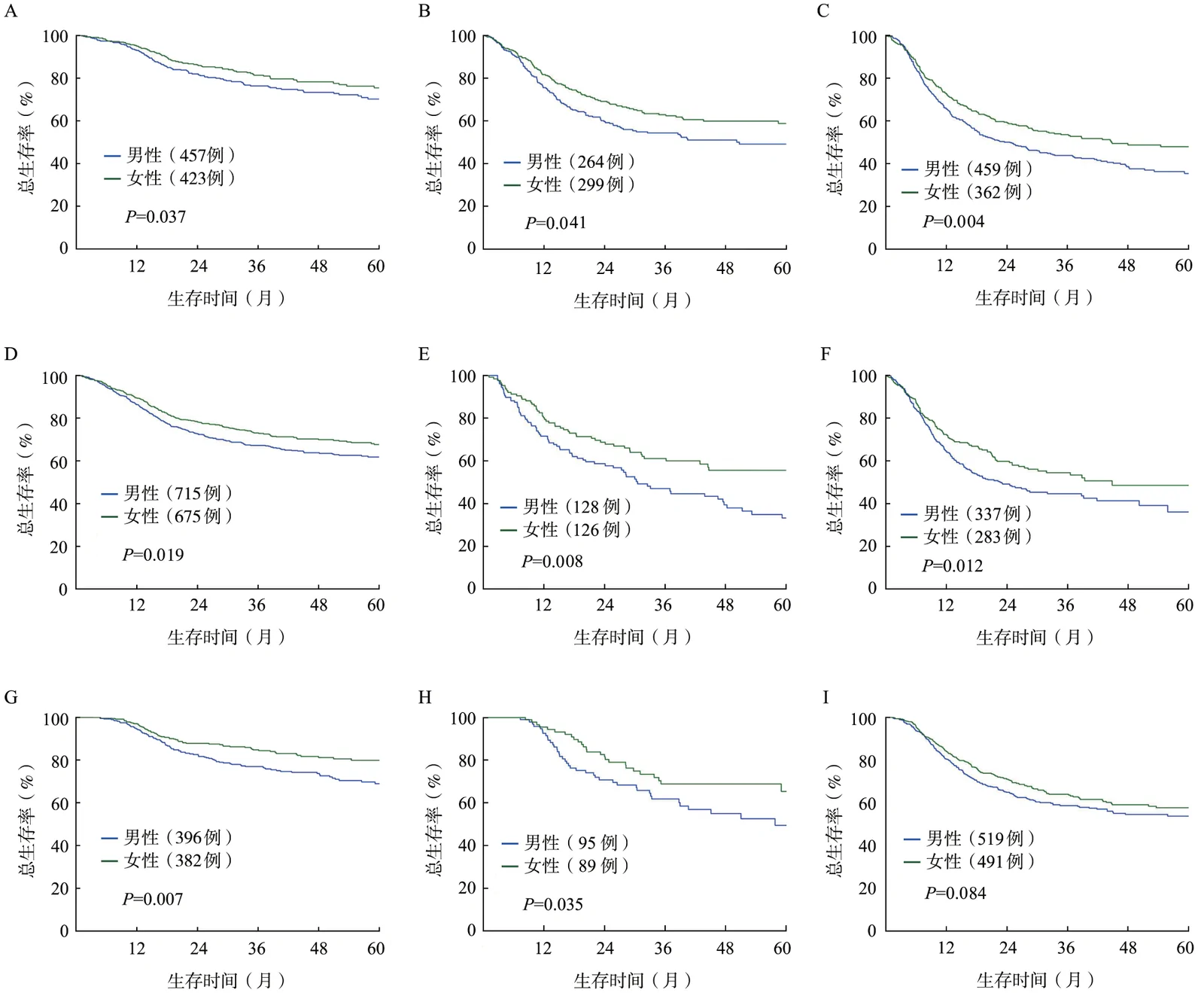
急性髓系白血病亚组中男性和女性的总生存比较 **A** ELN2022预后良好；**B** ELN2022预后中等；**C** ELN2022预后不良；**D** 强化疗；**E** CAG方案；**F** AZA-VEN方案；**G** CR_1_ allo-HSCT；**H** CR_2_或复发后未达CR/CRi allo-HSCT；**I** 非移植治疗 **注** ELN2022：2022年欧洲白血病网指南；强化疗：包括高三尖杉酯碱+阿糖胞苷+阿柔比星（HAA）、去甲氧柔红霉素+阿糖胞苷（IA）和柔红霉素+阿糖胞苷（DA）方案；CAG：阿糖胞苷+阿克拉霉素+G-CSF；AZA-VEN：阿扎胞苷+维奈克拉；CR_1_：第1次完全缓解；CR/CRi：完全缓解伴或不伴血液学恢复；allo-HSCT：异基因造血干细胞移植

预后中等的患者中，男性和女性的1个疗程CR/CRi率（63％对63％，*P*＝0.973）、最终CR/CRi率（86％对88％，*P*＝0.565）、4年CIR、累积NRM和RFS率差异均无统计学意义，但男性的4年OS率显著低于女性［51％（95％*CI*：45％～58％）对60％（95％*CI*：54％～66％），*P*＝0.041］（图3B）。多因素分析显示，男性与更低的OS率显著相关（*HR*＝1.33，95％*CI*：1.02～1.73，*P*＝0.035）。

预后不良的患者中，男性的1个疗程CR/CRi率（52％对60％，*P*＝0.020）和最终CR/CRi率（73％对80％，*P*＝0.041）较女性更低，男性的4年CIR和累积NRM与女性差异无统计学意义，而4年RFS率［42％（95％*CI*：36％～49％）对53％（95％*CI*：46％～60％），*P*＝0.026］和OS率［39％（95％*CI*：34％～44％）对49％（95％*CI*：43％～55％），*P*＝0.004］更低（图3C）。多因素分析显示，男性与更低的1个疗程CR/CRi率（*OR*＝0.72，95％ *CI*：0.53～0.96，*P*＝0.025）显著相关，有OS率更低的趋势（*HR*＝1.19，95％ *CI*：0.98～1.46，*P*＝0.079）。

3. 根据化疗方案分层：在接受强化疗（HAA、IA和DA）的患者（1 390例）中，男性和女性1个疗程CR/CRi率（70％对71％，*P*＝0.593）和最终CR/CRi率（91％对93％，*P*＝0.081）、4年CIR、累积NRM和RFS率差异均无统计学意义，男性的4年OS率［64％（95％*CI*：60％～68％）对70％（95％*CI*：66％～74％），*P*＝0.019］较女性显著更低（图3D）。多因素分析显示，男性比女性有OS率更低的趋势（*HR*＝1.18，95％ *CI*：0.98～1.41，*P*＝0.082）。

在接受CAG方案的患者（254例）中，男性和女性1个疗程CR/CRi率（54％对62％，*P*＝0.222）和最终CR/CRi率（77％对84％，*P*＝0.130）、4年CIR、累积NRM和RFS率差异均无统计学意义。男性患者的4年OS率［39％（95％*CI*：30％～49％）对56％（95％*CI*：46％～65％），*P*＝0.008］较女性显著更低（图3E）。多因素分析显示，男性与更低的OS率相关（*HR*＝1.56，95％ *CI*：1.05～2.31，*P*＝0.026）。

在接受AZA-VEN方案的患者（620例）中，男性的1个疗程CR/CRi率（62％对73％，*P*＝0.004）较女性更低，应用该方案期间男性的CR/CRi率（65％对75％，*P*＝0.011）亦较女性更低，但最终CR/CRi率（78％对80％，*P*＝0.567）差异无统计学意义。以初诊骨髓原始细胞的中位数52％为界，原始细胞<52％的患者中，男性的1个疗程CR/CRi率（59％对74％，*P*＝0.004）较女性更低；而原始细胞≥52％的患者中，男性和女性的1个疗程CR/CRi率差异无统计学意义（66％对72％，*P*＝0.311）。4年CIR、累积NRM和RFS率差异无统计学意义，而男性患者的4年OS率［41％（95％*CI*：35％～48％）对49％（95％*CI*：41％～57％）,*P*＝0.012］较女性显著更低（图3F）。多因素分析显示，男性与更低的1个疗程CR/CRi率相关（*OR*＝0.62，95％ *CI*：0.43～0.89，*P*＝0.009），有OS率更低的趋势（*HR*＝1.24，95％ *CI*：0.97～1.58，*P*＝0.078）。

4. 根据移植分层：在778例CR_1_接受移植的患者中，男性和女性患者的4年CIR和累积NRM差异无统计学意义。男性的4年RFS率［70％（95％*CI*：65％～75％）对76％（95％*CI*：71％～81％），*P*＝0.049］和4年OS率［74％（95％*CI*：69％～78％）对81％（95％*CI*：77％～85％），*P*＝0.007］均低于女性（图3G）。复发患者中，男性的4年OS率［25％（95％*CI*：14％～35％）对41％（95％*CI*：27％～55％），*P*＝0.032］低于女性。多因素分析显示，男性与更低的RFS率（*HR*＝1.37，95％ *CI*：1.02～1.82，*P*＝0.035）和OS率（*HR*＝1.53，95％ *CI*：1.12～2.10，*P*＝0.008）相关。在184例CR_2_或复发后未达CR/CRi接受移植的患者中，男性的4年OS率［54％（95％*CI*：43％～66％）对69％（95％*CI*：58％～80％），*P*＝0.035］亦显著低于女性（图3H），多因素分析显示，男性与更低的OS率相关（*HR*＝2.31，95％ *CI*：1.33～4.01，*P*＝0.003）。

在未接受移植的1 010例患者中，男性的4年CIR、NRM与女性差异无统计学意义，4年RFS率［50％（95％*CI*：45％～55％）对52％（95％*CI*：47％～57％），*P*＝0.164］和OS率［55％（95％*CI*：49％～60％）对59％（95％*CI*：54％～64％），*P*＝0.084］均有低于女性的趋势（图3I），多因素分析未发现性别与结局相关。

## 讨论

本研究对中国AML患者男性和女性之间的临床和分子遗传学特征、治疗反应和结局进行了深入分析。与国外的报道相似，本研究发现男性的CR/CRi率、RFS率和OS率均更低[5]。在2项国外的AZA-VEN治疗AML患者的研究中，男性的CR/CRi率比女性低，分别为59％对66％（*P*＝0.19）和58％对66％（*P*＝0.13），但差异均无统计学意义[13]–[14]。本研究首次在中国人群中验证了男性对于AZA-VEN治疗的1个疗程CR/CRi率和最终CR/CRi率均显著低于女性，该差异在低骨髓原始细胞比例人群更显著，接受强化疗挽救治疗后男性和女性的CR/CRi率相似。

男性和女性的预后差异，与男性更常携带骨髓增生异常相关的基因突变和细胞遗传学异常相关。Ozga等[5]的研究发现，男性的不良结局仅存在于ELN2022预后不良组的患者。本研究发现，预后不良的患者中男性CR/CRi率、RFS率和OS率均低于女性，预后良好/中等的患者中男性和女性的CR/CRi率和复发率相似，但男性的OS率更低。该结果提示男性仍存在分子遗传学以外的因素导致不良预后。

药物代谢的性别差异是一个影响预后的潜在重要因素。药代动力学研究发现男性对多柔比星的清除率更高，而柔红霉素的清除未见明显性别差异，但是随着柔红霉素剂量增加，女性的生存获益大于男性[5],[15]–[16]。维奈克拉在女性的中心室分布容积较男性低28％～32％，但男性和女性对维奈克拉的清除率相似，因此不支持根据性别调整剂量[17]。胞苷脱氨酶是胞苷类似物的清除中核心的代谢酶，其活性是骨髓增生异常肿瘤（MDS）患者的独立预后生物标志物：活性越低，生存期越长[18]。男性的胞苷脱氨酶活性更高，导致药物半衰期缩短，这可能是男性MDS患者应用去甲基化药物生存更差的原因，且由于耗竭DNA甲基转移酶1的作用依赖于DNA复制期（S期），在低S期比例的患者中男女的生存差异更大[19]。本研究中应用AZA-VEN的患者中男性的1个疗程CR/CRi率显著低于女性，且该差异仅在原始细胞<52％的人群中显著，提示AZA代谢的性别差异可能解释了疗效差异的部分原因。

在接受移植的患者中分析男性和女性的预后差异，不论是CR_1_还是CR_2_或未达CR时接受移植，男性的RFS率和OS率都更低，复发后的生存差异是主要的差异来源。另外，本研究发现移植患者中男性的NRM有高于女性的趋势，其原因可能是女供男的移植较其他供受者组合的移植物抗宿主病和移植相关死亡风险更高，该现象的可能机制是男性受者接受女性的造血干细胞后产生了Y染色体编码次要组织相容性抗原的同种异体抗体[20]。

由于本研究为单中心回顾性研究，治疗方案存在异质性，随访时间较短，亚组分析的样本量有限，本研究的结论需要在更大样本的多中心研究中验证。

综上，在AML患者中，与女性相比，男性除了HGB、尿酸和白蛋白水平更高和估算肾小球滤过率更低以外，更常携带预后不良的基因突变和染色体异常，治疗反应率和OS率更低。男性患者对AZA-VEN方案的治疗反应率更低，且在低骨髓原始细胞的患者中差异显著；接受移植的患者中男性的生存率更低。
